# Single-cell transcriptome conservation in a comparative analysis of fresh and cryopreserved human skin tissue: pilot in localized scleroderma

**DOI:** 10.1186/s13075-020-02343-4

**Published:** 2020-11-09

**Authors:** Emily Mirizio, Tracy Tabib, Xiao Wang, Wei Chen, Christopher Liu, Robert Lafyatis, Heidi Jacobe, Kathryn S. Torok

**Affiliations:** 1 Division of Rheumatology, Department of Pediatrics, Children’s Hospital of Pittsburgh, University of Pittsburgh, Pittsburgh, PA USA; 2 Division of Rheumatology, Department of Medicine, University of Pittsburgh, Pittsburgh, PA USA; 3 Division of Pediatric Pulmonary Medicine, UPMC Children’s Hospital of Pittsburgh, University of Pittsburgh, Pittsburgh, PA USA; 4 Department of Dermatology, University of Texas Southwestern Medical Center, Dallas, TX USA; 5 University of Pittsburgh Medical Center Children’s Hospital of Pittsburgh Faculty Pavilion, 3rd floor, Office 3117 4401 Penn Avenue, PA 15237 Pittsburgh, USA

**Keywords:** Localized scleroderma, Morphea, Single-cell RNA sequencing, Pediatric rheumatology, Cryopreservation, Transcriptome expression

## Abstract

**Background:**

The purpose of this study was to assess variability in cell composition and cell-specific gene expression in the skin of patients with localized scleroderma (LS) utilizing CryoStor® CS10 in comparison to RPMI to produce adequate preservation of tissue samples and cell types of interest for use in large-scale multi-institutional collaborations studying localized scleroderma and other skin disorders.

**Methods:**

We performed single-cell RNA sequencing on paired skin biopsy specimens from 3 patients with LS. Each patient with one sample cryopreserved in CryoStor® CS10 and one fresh in RPMI media using 10× Genomics sequencing.

**Results:**

Levels of cell viability and yield were comparable between CryoStor® CS10 (frozen) and RPMI (fresh) preserved cells. Furthermore, gene expression between preservation methods was collectively significantly correlated and conserved across all 18 identified cell cluster populations.

**Conclusion:**

Comparable cell population and transcript expression yields between CryoStor® CS10 and RPMI preserved cells support the utilization of cryopreserved skin tissue in single-cell analysis. This suggests that employing standardized cryopreservation protocols for the skin tissue will help facilitate multi-site collaborations looking to identify mechanisms of disease in disorders characterized by cutaneous pathology.

## Background

Technological advancements in the last 20 years have allowed for a deeper understanding of complex organ systems via exploration of cell-type composition and transcriptome heterogeneity on a single-cell level. Analysis at this level of complexity has been difficult to overcome and many technologies have evolved with differing levels of granularity [1–3]. One of the most comprehensive techniques for this analysis is single-cell RNA sequencing (scRNAseq) which provides transcriptional data for each cell in tissue samples composed of mixed cell populations [4–6]. This allows for cell-type-specific analysis of transcriptomic expression for multiple types of disease investigation in tissue, such as baseline characterization of cellular immunophenotype, disease development and progression, and treatment effects.

Our laboratory has an interest in the examination of the transcriptome in the localized scleroderma (morphea) skin using a high-resolution scRNAseq technology with advanced bioinformatics to better characterize the dysregulation of the IFN-γ pathway and contributing cell types that are likely relevant to disease propagation [7–12]. To study the skin transcriptomic immunophenotype across the clinical spectrum of localized scleroderma (LS), we formulated a dermatology-rheumatology partnership across two academic centers with robust LS-specific cohorts. These registries, the National Registry of Childhood Onset Scleroderma (NRCOS) and the Morphea in Adults and Children (MAC), provide a rich resource of well-phenotyped patients interested in providing skin biopsy samples for our research protocols. Before undertaking a large and expensive scRNAseq study across two physically distant academic centers, we performed a pilot study to ensure the integrity of skin cellular specimen preservation. The overall intention is that these validation steps for proper sample procurement of skin for scRNAseq will not only set the stage for our larger study in localized scleroderma (morphea), but also serve as a platform for technical reference and cell type preservation for other rare autoimmune skin diseases, such as cutaneous lupus erythematosus and dermatomyositis, in which obtaining materials across multiple centers as a consortium would be beneficial.

Proper sample preparation in scRNAseq studies is paramount for comprehensive identification and analyses of the variety of cell types in any tissue being examined, including the skin. Viable and fully disassociated samples from any tissue sources are essential for proper cell loading into scRNAseq systems [13–16]. Because of the need for viable single-cell suspensions for scRNAseq analysis, sample preservation methods are limited, so fresh samples are preferred. Given the challenges of solely using fresh tissue, one method of interest to preserve cells and tissue samples is cryopreservation. However, freeze-thaw cycles have adverse effects on many tissues and can cause cell membrane rupture; molecular damage to DNA, proteins, and lipids; and denaturing of proteins from freeze concentration of standard buffers [17, 18]. Additionally, heterogeneous cell types react differently to freezing techniques so certain populations of cells could be lost in the freeze-thaw process [19]. We identified a well-validated method to circumvent these adverse effects that uses cryoprotectants, which act intracellularly to prevent crystallization and cell rupture, and extracellularly to reduce hyperosmotic effects that cause denaturation [19, 20]. Common cryoprotectants include dimethylsulfoxide (DMSO), glycerol, and ethylene glycol which act intracellularly, and sucrose, dextrose, and polyvinylpyrrolidone which act extracellularly [17, 19]. All of these agents mitigate freeze-thaw effects but do not solve the issue of cell loss entirely. Cryopreservation of samples was of obvious interest to our localized scleroderma group in which we require specimens collected from remote sites to achieve adequate power for the study of this rare disease. However, little data is available on the effect of cryopreservation on skin specimens.

A number of cryoprotectants have been evaluated for scRNAseq applications in other tissues. CryoStor® CS10 is a popular cryopreservation solution produced by BioLife Solutions® with both intracellular and extracellular cryopreservation agents (proprietary combination including DMSO (10%) and sucrose) to efficiently freeze samples for downstream use [20, 21]. CryoStor® CS10 has been used to preserve synovial and renal tissue samples for scRNAseq in a large multicenter program, the Accelerating Medicines Partnership RA/SLE Network (AMP) [22–24], and demonstrated replicable cell populations and transcript expression compared to fresh samples in synovial and renal tissue shipped to a central processing site [22–24]. However, AMP has not been evaluated in the preservation of skin cell types with scRNAseq. Given CryoStor® CS10’s validated use as a cryoprotectant in multi-center studies examining other tissues, we undertook a pilot study to determine its performance in the skin from patients with localized scleroderma collected across two LS centers. Our objective was to determine whether the use of CryoStor® CS10 produced adequate preservation of tissue samples and the cell types of interest allowing for the use of frozen samples for large-scale studies in localized scleroderma skin, as well as other skin disorders that benefit from multi-institutional collaborations.

## Methods

### Human patient samples

Developed in 2003 and 2007, respectively, the NRCOS (University of Pittsburgh, PI - Torok) and MAC (University of Texas Southwestern, PI – Jacobe) cohorts collect and link patient data with biological specimens through well-developed, standardized methods for recruitment, data capture, data management, and handling of biological specimens, with the personnel and infrastructure to support these activities [8, 10–12, 25–28]. Research participants in both cohorts are required to meet the diagnostic criteria of LS [29] and undergo an IRB-approved consent process to collect predefined case report forms and biospecimens (blood, skin, saliva) [8, 10–12, 25–28]. Pediatric-onset disease is defined as onset at < 18 years of age. Participants enrolled in either cohort that meet inclusion criteria are eligible for the collection of skin for research purposes. For this pilot study, an additional IRB-approved consent was obtained for the collection of two 3-mm-punch biopsies of the areas affected by LS from adult patients in the MAC cohort.

### Skin biopsy collection and shipment

Three LS subjects with lesional biopsy specimens were analyzed, each with a 3-mm-punch biopsy preserved in CryoStor® CS10 (frozen) and an adjacent 3-mm-punch biopsy preserved in RPMI (fresh). The average depth of the biopsies was 3.4 mm. These two biopsies were allocated for scRNAseq; one whole biopsy was placed in Roswell Park Memorial Institute (RPMI) 1640 Medium (Gibco®, Gaithersburg, MD) and put on ice while the other whole biopsy was placed in chilled CryoStor® CS10 cell preservation media (BioLife Solutions®, Bothell, WA), incubated at 4 °C for 10 min then put on dry ice per manufacturer’s instructions. Samples were then shipped overnight via FedEx for scRNAseq processing and analysis at the University of Pittsburgh.

### Skin processing and single-cell RNA sequencing

Upon arrival at the University of Pittsburgh, Single Cell Genomics Core, Sequencing Facility, sample dissociation and processing was performed identically. Samples were thawed or kept on ice (dependent on shipping type) before being enzymatically digested (Miltenyi Biotec Whole Skin Dissociation Kit, human) for 2 h and further dispersed using the Miltenyi gentleMACS Octo Dissociator. The resulting cell suspension was filtered through 70-μm cell strainers twice and re-suspended in PBS containing 0.04% BSA.

Cells from biopsies were mixed with reverse transcription reagents then loaded into the Chromium instrument (10× Genomics), a commercial application of Drop-Seq [30]. This instrument separated cells into mini-reaction “partitions” formed by oil micro-droplets, each containing a gel bead and a cell, known as Gel Bead-In-Emulsions (GEMs). GEMs contain a gel bead, scaffold for an oligonucleotide that is composed of an oligo-dT section for priming reverse transcription, and barcodes for each cell (10×) and each transcript (unique molecular identifier, UMI), as described [31]. Approximately 1000-fold excess of partitions compared to cells ensured low capture of duplicate cells. ~ 2600–4300 cells were loaded into the instrument to obtain data on ~ 1100–2300 cells, anticipating a multiplet rate of ~ 1.2% of partitions. V2 single-cell chemistries were used per manufacturer’s protocol (10× Genomics).

Briefly, the reaction mixture/emulsion was removed from the Chromium instrument, and reverse transcription performed. The emulsion was then broken using a recovery agent, and following Dynabead and SPRI clean up, cDNAs were amplified by 11–12 cycles of PCR (C1000, Bio-Rad). cDNAs were sheared (Covaris) into ~ 200 bp length. DNA fragment ends were repaired, A-tailed, and adaptors ligated. The library was quantified using KAPA Universal Library Quantification Kit KK4824 and further characterized for cDNA length on a Bioanalyzer using a High Sensitivity DNA kit. RNA-seq. Libraries were sequenced (~ 200 million reads/sample), using the Illumina NextSeq-500 platform.

### Data preprocessing and bioinformatics analysis

Sequencing reads were examined by quality metrics, transcripts mapped to reference human genome (GRCh38), and assigned to individual cells according to cell barcodes, using Cell Ranger (10× Genomics). Data from the study is deposited on NCBI Gene Expression Omnibus (GSE160536).

Further data analysis was performed using R (version 3.5), specifically the Seurat 3.0 package for normalization of gene expression and identification and visualization of cell populations [32, 33]. Briefly, the UMI matrix was filtered such that only cells expressing at least 200 genes were utilized in downstream analysis. Unwanted sources of variation were regressed out of the data by constructing linear models to predict gene expression based on the number of UMIs per cell as well as the percentage of mitochondrial gene content. Additional filtering was applied to tables examining differential gene expression between cells or groups of cells within clusters, by filtering cells out that were expressed in less than 10% of the cells showing upregulated expression.

The data was further normalized between samples using SCTransform which models technical noise using a regularized negative binomial regression model [34]. Principal component analysis (PCA) was subsequently performed on the scaled data of the identified highly variable genes.

Finally, cells were clustered using a smart local moving algorithm (SLM). Cell populations were identified based on gene markers in the associated transcriptomes and visualized by t-distributed stochastic neighbor embedding (t-SNE) [35]. Out of 33,538 detected genes, 5000 were determined to be variable between identified clusters. AddModuleScore was utilized to calculate the average expression levels of each input (either gene or cluster) on a single-cell level, subtracted by the aggregated expression of control feature sets.

### Statistical analysis

Differential gene expression between sample types was assessed using Seurat’s implementation of the non-parametric Wilcoxon rank-sum test and a Bonferroni correction was applied to the results. Spearman’s correlations and other statistical tests run in R utilized the packages devtools and ggplot. GraphPad Prism version 8.0 was used to compare cellular populations between processing types using Wilcoxon matched-pairs signed-rank tests.

## Results

### Cellular retrieval and transcriptomic expression of the cryopreserved skin is comparable to the fresh skin

**Fig. 1:**
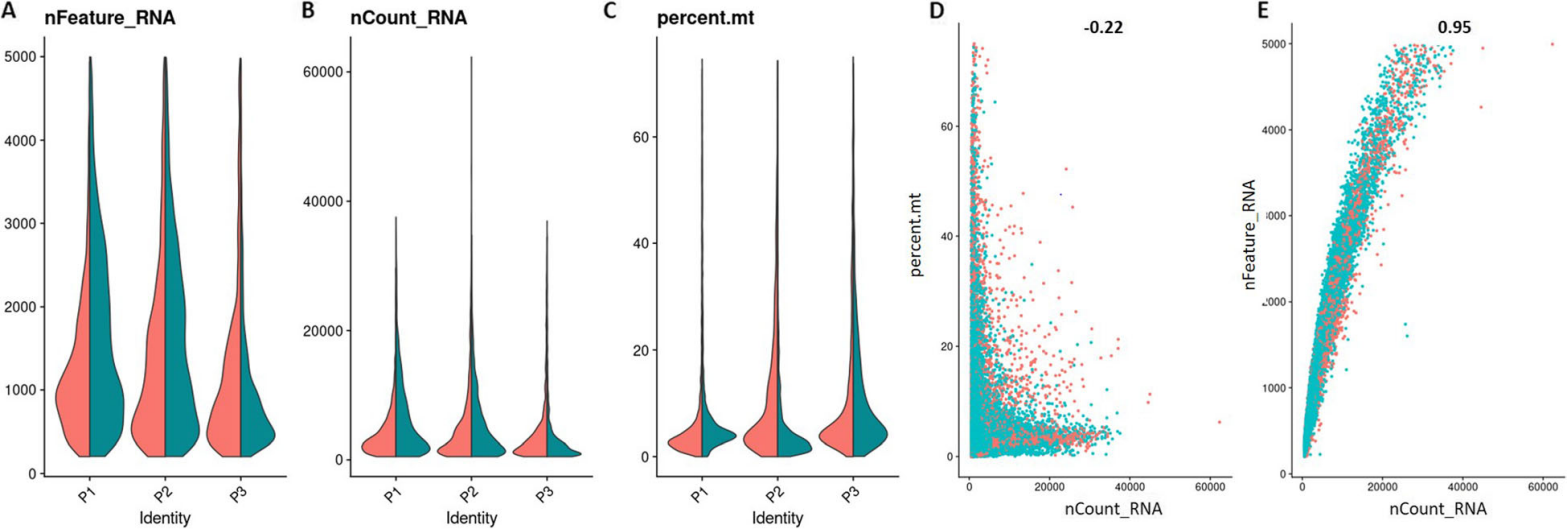
Quality control (QC) metrics of single-cell data between paired cryopreserved (CryoStor CS10®, pink) and fresh (RPMI, blue) samples demonstrate equivalence between methods. QC metrics, including **a** the number of unique genes, **b** the number of total molecules, and **c** the percentage of reads that map to the mitochondrial genome, all demonstrate equivalence between preservation methods, with **d** low correlation of mitochondrial genes across cells and **e** high correlation of gene expression across cells. Three patients are shown (P1, P2, and P3), with paired frozen (pink) and fresh (blue) skin samples

Cell viability and yield parameters comparing CryoStor® CS10 and RPMI preservation methods demonstrated 60–75% and 70–80% viability, with 6242 and 8659 total cells remaining after digest, respectively (Table 2). ScRNA seq performance metrics, such as the read quality and sequencing saturation, were equivalent to fresh samples at 80–90% and 70–90%, respectively (Table 1). The number of feature genes, RNA, and mitochondrial DNA was also analogous between sample types (CryoStor® and RPMI) for each individual patient sample and collectively (Fig. 1). The Spearman’s correlation coefficient was 0.95 (*p* <  0.0001) when comparing between the number of unique RNA features (nFeature) detected in each cell and the total number of UMI counts within a cell across all cells (nCounts) (Fig. 1). Analysis of the raw data before normalization supports these same findings (Supplemental Figure 1).

**Table 1:** Single-cell sequencing performance metrics are equivalent between skin specimens preserved in frozen media (CryoStor® CS10) compared to fresh media (RPMI)

Patient	Preservation method	Mean reads per cell	Median genes per cell	Sequencing saturation (%)
P1	CryoStor	93,607	1023	89
	RPMI	52,979	1353	76
P2	CryoStor	114,765	1135	89
	RPMI	53,131	1410	75
P3	CryoStor	104,331	896	87
	RMPI	163,811	710	94

### Eighteen distinct cell clusters were identified and shared in both frozen and fresh preservation methods

**Fig. 2:**
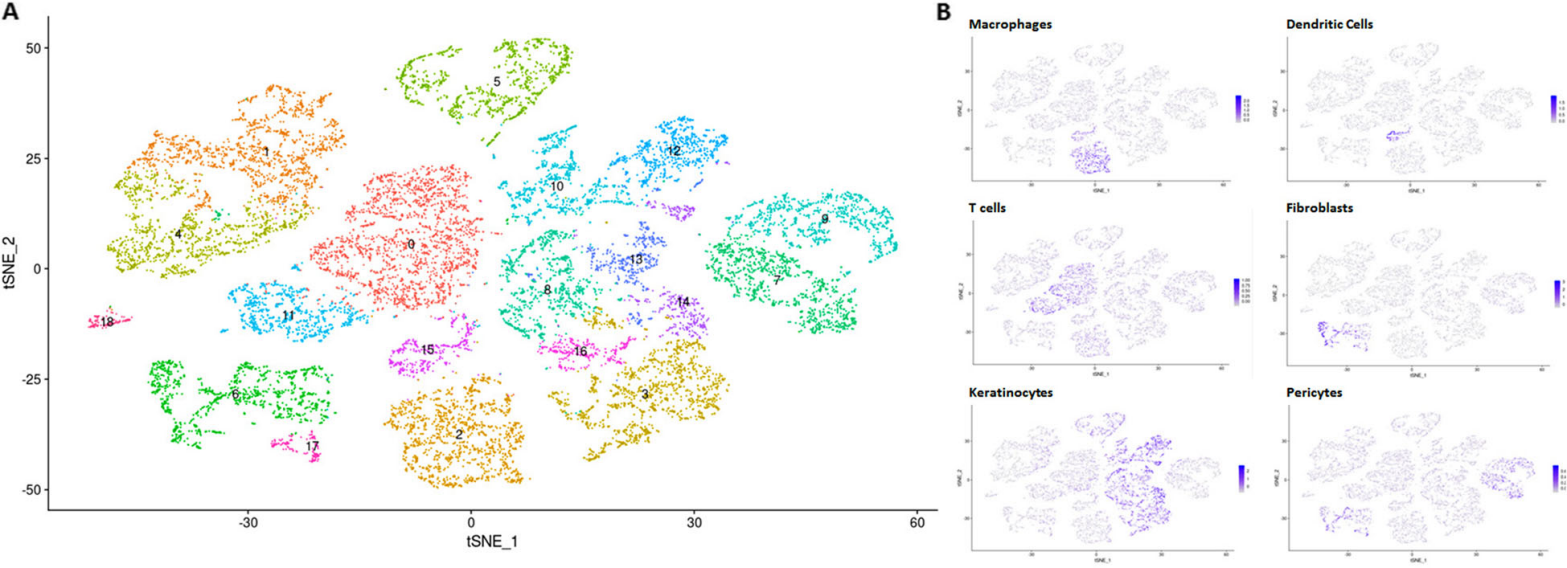
The t-distributed stochastic neighbor embedding (t-SNE) plot of the 6 skin samples (3 sets of paired fresh and frozen) demonstrates 18 cell clusters. **a** Gene expression profiling of known cell types was used to define cell clusters. **b** Gene signatures *(as listed in text)* confirm main cell types identified via feature plots (right)

Clustering analysis, using an unsupervised graph-based clustering algorithm, of 14,901 cells from 3 patients with 1 fresh and 1 frozen sample each (6 total samples) identified eighteen distinct clusters of cells displayed by color on the t-distributed stochastic neighbor embedding (t-SNE) plot in Fig. 2a. These cell clusters were identified by comparing overall gene expression to well-known expression profiles of cell types in the skin by calculating the differential expression between clusters. Keratinocytes were identified by cell cluster expression of KRT1, KRT14, KRT17, and KRT15; pericytes by RGS5, CSPG4, NG2, PDGFRB, and RERGL; T cells by CD3D, CD3E, CD8A, FOXP3, CD4; macrophages by CD163 and AIF1; dendritic cells (DCs) by CD1C, FCER1A, and SPP1; fibroblasts by COL1A1, COL1A2, PDGFRA, FMO1, MYOC, and SFRP2; myofibroblasts by WIF1, NKD2, PCOLCE2, SLP1, CD55, ACTA2, WNT2, SMA; smooth muscle cells by DES and SMTN; natural killer (NK) cells by NKG7; melanocytes by PMEL; B cells by IGJ and MS4A1; secretory (glandular) cells by CA6 and SCGB1B2P; mast cells by TPSAB1; and endothelial and lymphatic endothelial cells by VWF, CLDN5, CDH5, and LYVE1 (Fig. 2). These and other markers provided strong transcriptome signatures for each cell cluster and identified a total of 9 main cell groupings from the overall 18 clusters, listed in Table 2 and demonstrated with feature plots in Fig. 2b and heat map in Supplementary Figure 2.

**Table 2:** Total number of cells recovered and cell type recovery overall is comparable between preservation methods in frozen media (CryoStor® CS10) compared to fresh media (RPMI) in the 18 cell clusters within the 9 cell groupings

Subject and preservation method		P1 CryoStor	P1 RPMI	P2 CryoStor	P2 RPMI	P3 CryoStor	P3 RPMI	Avg. % cell type per preservation	
Total number cells recovered		2576	3549	1707	3846	1959	1274	CryoStor	RPMI
Main cell grouping	Clusters								
T/NK cell (%)	0, 11	37	19	6	14	15	2	22	14
Epithelial/RBC (%)	1, 4	18	5	19	13	25	40	20	14
Macrophages (%)	2	24	15	0	1	0	0	10	7
Keratinocytes (%)	3, 8, 10, 12, 13, 14, 16	7	48	26	31	22	32	17	38
Epithelial (%)	5	1	1	24	10	5	6	8	5
Fibroblast (%)	6, 17	5	6	5	14	7	0	6	9
Pericyte (%)	7, 9	6	3	14	13	22	19	13	10
Dendritic cells (%)	15	2	2	4	3	3	0	2	2
Lymphatic endothelial (%)	18	1	1	1	1	1	0	1	1

**Fig. 3:**
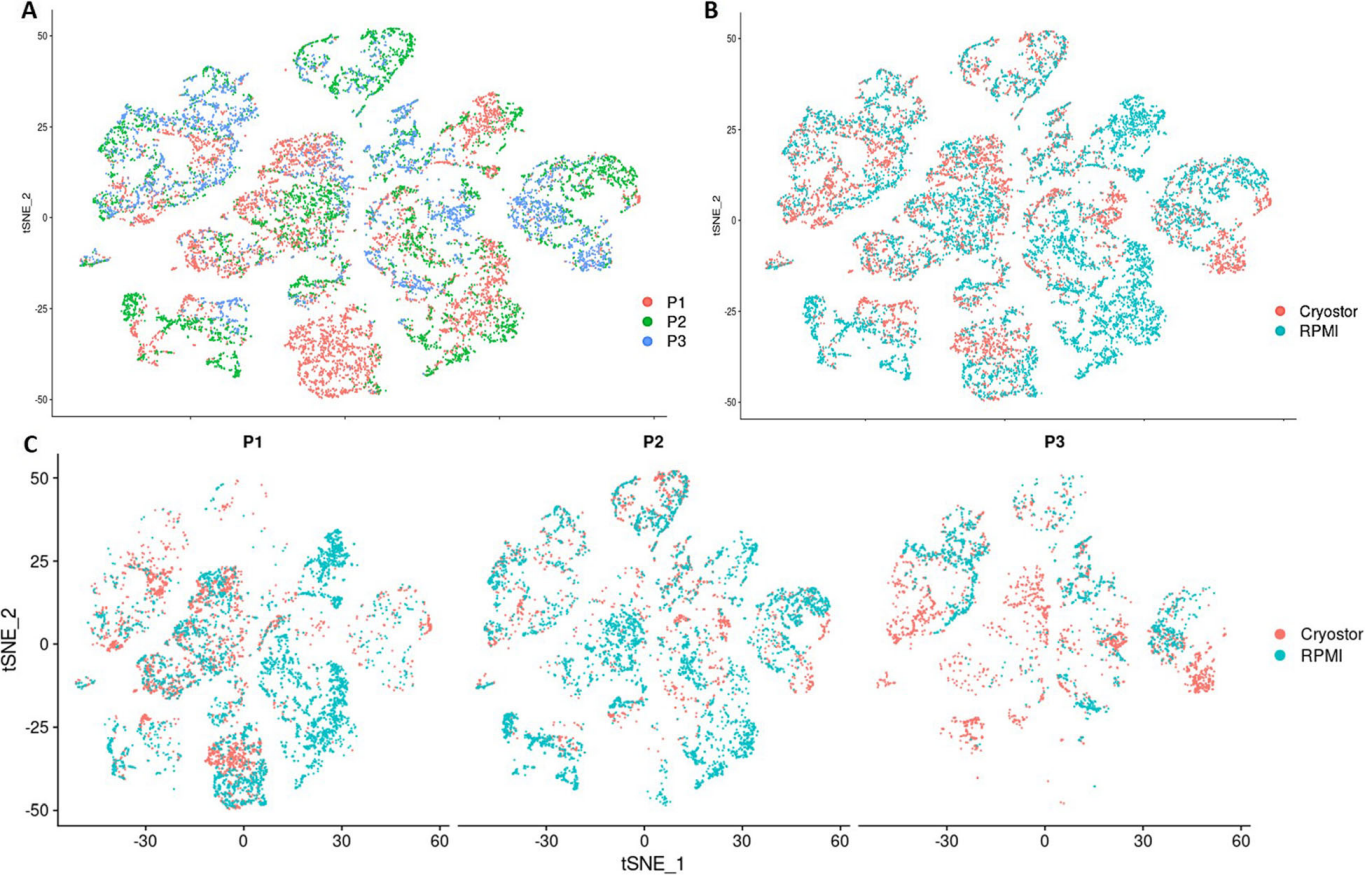
Detailed t-distributed stochastic neighbor embedding (t-SNE) plots comparing transcriptomic expression between the three patients (P1, P2, and P3) and the preservation method (CryoStor® vs. RPMI) demonstrate even dispersion among cell clusters. **a** Three patients overlap well with cellular transcriptomic expression across the 18 cell clusters. **b** CryoStor**®** (frozen) and RPMI (fresh) preservation methods show even dispersion across cell clusters. **c** Individual patient with paired frozen and fresh specimens demonstrate even dispersion. These t-SNE plots represent 14,901 skin cells, derived from 3 patients with localized scleroderma (3 cryopreserved and 3 fresh samples with 6242 and 8659 cells, respectively)

Each of the 18 clusters, which compose 9 main cell groupings, included cells from each biopsy sample and preservation type (fresh vs. frozen) as demonstrated in Fig. 3, supporting the overall conservation of cell types in frozen preserved samples. Analysis of the raw data before normalization supports these same findings of even disbursement (Supplemental Figure 3). The total number of cells extracted from CryoStor® CS10 preserved samples was 72% of that extracted from fresh samples (Table 2). Cell types most affected were keratinocytes, with an average 21% loss of total number via cryopreservation, and the remainder differences of other cell types were relatively negligible, having only 7% or less cell loss with cryopreservation (Table 2). Despite the percentage of cell lost per cell type, statistical grouped analysis of cell populations did not show any statistical difference between preservation techniques using Wilcoxon rank testing (Supplementary Table 2).

### RNA expression is conserved across cell populations using CryoStor® skin preservation

**Fig. 4:**
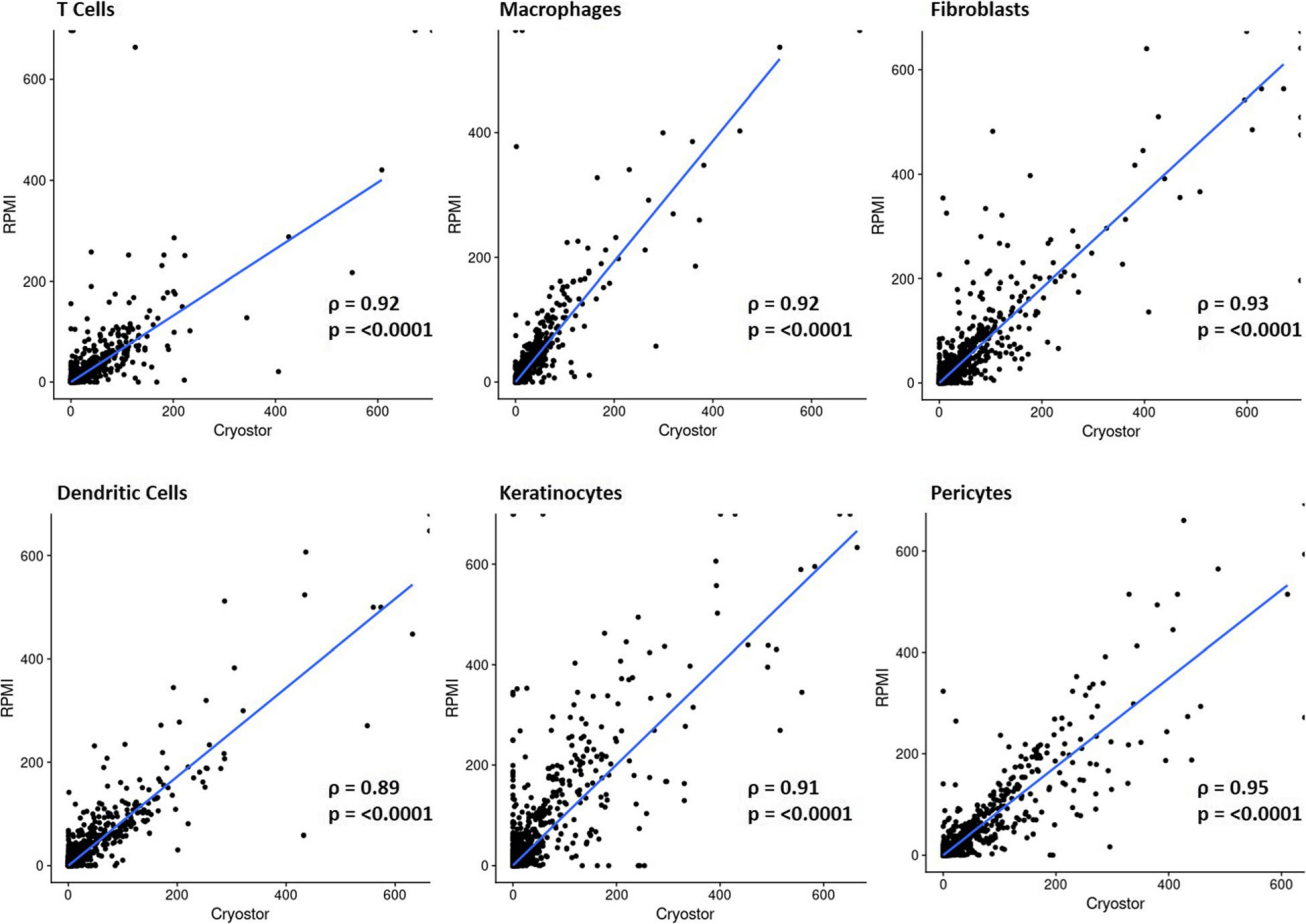
Correlation of average genetic expression for major cell groups demonstrates a high correlation between sample preservation types. Fresh and cryopreserved samples correlated highly and significantly within cell groups including T cells, macrophages, fibroblasts, dendritic cells, keratinocytes, and pericytes. Each point on the correlation plots display the average UMI counts for each gene across all cells for each major cell group

The average expression of genes for major cell groups, such as keratinocytes, T/NK cells, DC/macrophages, fibroblasts, and pericytes, demonstrated a strong correlation (*r*_*S*_ > 0.90, *p* <  0.0001) between the average UMI counts for each gene across all cells in the respective group (Fig. 4, Table 3). Analysis of the raw data before normalization supports similar correlation coefficients (Supplemental Table 1, Supplemental Figure 4). Differential expression analysis between the three CryoStor® CS10 and three fresh sample types revealed only 123 differentially expressed genes between processing types, with only a maximum of 1.2 log fold change (Supplement Table 3). Compared to the 15,375 genes analyzed (after quality and representation trimming), this is only 0.8% of genes that are differentially expressed. The majority of these genes had lower expression in the frozen (CryoStor) specimens compared to the matched fresh (RPMI), with genes related to known cell type losses, such as keratinocytes (KRT1, KRT2, KRT10) and adipose cells (AQP3), in the frozen tissue (Table 4, Supplement Table 3).

**Table 3:** Transcriptomic expression of genes within cell types was similar between preservation methods in frozen media (CryoStor® CS10) compared to fresh media (RPMI). Strong spearman’s correlation coefficients were demonstrated among all cell types

	Spearman’s Rho	*p* value
T cells	0.92	< 0.0001
Macrophages	0.92	< 0.0001
Dendritic cells	0.89	< 0.0001
Keratinocytes	0.91	< 0.0001
Fibroblasts	0.93	< 0.0001
Pericytes	0.95	< 0.0001

**Table 4:** Top 10 differentially expressed genes comparing paired frozen media (CryoStor® CS10) compared to fresh media (RPMI) skin samples. *Full 123 DEGs are in Supplement*

	Average log fold change	***p*** value	% of cells in PCA 1	% of cells in PCA 2	Adjusted p value
*KRTDAP*	− 1.08932	< 0.0001	0.053	0.302	< 0.0001
*DSC3*	− 0.53179	< 0.0001	0.06	0.295	< 0.0001
*S100A14*	− 0.70139	< 0.0001	0.086	0.33	< 0.0001
*PERP*	− 1.00375	< 0.0001	0.4	0.635	< 0.0001
*DSP*	− 0.89602	< 0.0001	0.13	0.381	< 0.0001
*SERPINB2*	− 0.5951	< 0.0001	0.026	0.23	< 0.0001
*AQP3*	− 0.83687	< 0.0001	0.145	0.388	< 0.0001
*LY6D*	− 0.72067	< 0.0001	0.08	0.319	< 0.0001
*SERPINB5*	− 0.45845	< 0.0001	0.054	0.273	< 0.0001
*KRT2*	− 1.12371	< 0.0001	0.017	0.206	< 0.0001

## Discussion

Here, we demonstrate that CryoStor® CS10 preservation is an acceptable alternative to fresh tissue for skin biopsy specimen preparation for single-cell sequencing, which has the potential to facilitate multi-center clinical trials and research of skin disease across institutions. We found immune cells (T cells, macrophages, NK cells) and fibroblasts recover well with this preparation and are of interest for our collaborative efforts in localized scleroderma (morphea) research. We also found gene expression from these cell types closely mirrored each other whether prepared using fresh (RPMI) or CryoStor® CS10 methodologies. The cellular and transcriptomic preservation data in our study of localized scleroderma skin reflects findings of other tissue types using CryoStor® CS10 including kidney [24], synovial tissue [23], peripheral blood mononuclear cells (PBMC), ovarian tumor, and colon tissue [36]. Despite differences in cell viability, both fresh and cryopreserved samples had comparable numbers of sequencing reads and detected genes in these other tissue types [22, 24, 36]. Dimensionality reduction via PCA and t-distributed stochastic neighbor embedding representations (t-SNE) shows the similarity between fresh and cryopreserved samples [22, 24, 36]. In addition to proving to be a viable methodology for preserving cell type and transcripts, employing a freezing strategy will enable investigators to collect more samples without being dependent on immediate processing of tissue for library preparation, which is a time-sensitive process requiring extensive technician time. It also permits sample digest and chip preparation of multiple samples together on the same cycle which minimizes potential batch effects that could mask subtle gene expression signatures and substantially decreases cost.

One limitation of cryopreservation in the skin is its effect on keratinocyte populations, both in our study and in a recent manuscript of scRNA seq cryopreserved skin in atopic dermatitis, which compared one fresh and cryopreserved healthy skin sample [37]. Their comparison demonstrated a lack of specific keratinocyte populations, including stratum corneum cells expressing late differentiation markers (e.g., FLG), but seemed to have better recovery of some fibroblast and vascular endothelial cell populations [37]. These findings were mirrored in our own data when similar profiling was used (Supplement, Figure S5-S6). Our data suggests negligible differences in these cell types with minimal cell loss and correlated expression. However, our data is limited in the exploration of keratinocyte subpopulations due to the sample size of 3 participants (6 paired samples) and samples being all localized scleroderma. The preservation of keratinocyte populations may be more essential in skin diseases with known epidermal pathophysiology, such as psoriasis. In addition, skin diseases, like localized scleroderma (morphea) and systemic sclerosis, which result in the inflammatory infiltrate and collagen deposition in the dermis (deeper skin pathology), may have originating pathophysiology from epidermal-dermal communication via keratinocyte activation through signaling channels such as WNT pathways [38].

Overall, we have demonstrated that cryopreservation of the skin with CryoStor® CS10 obtains equivalent yield of cell populations and cell expression of transcripts compared to fresh skin scRNA seq analyses for immune cells, fibroblasts, and endothelial cells, but may negatively affect certain clusters of keratinocyte and adipocyte populations. Further validation of keratinocyte population preservation may be warranted for skin diseases of known keratinocyte pathophysiology. The quality of preservation exhibited is on par with that demonstrated by the AMP project studying CryoStor® CS10 in synovial and renal tissue. Specifically for our disease of interest, localized scleroderma, with the current understanding of pathogenesis supported by an interaction of macrophage, T cell, fibroblast, and endothelial cell populations, which were all highly conserved, we will proceed with our larger project evaluating these cells and their transcripts using CryoStor® CS10 across institutions.

## Conclusion

This pilot study demonstrates the feasibility and potential of analyzing viable cells from cryopreserved skin tissue, specifically using the preservation solution CryoStor® CS10, which is the solution used for synovial and renal tissue cryopreservation for the large multi-centered collaborative AMP project. Using standardized skin processing protocols of these cryopreserved samples at a single-cell sequencing core provides high yields of viable cells with preserved transcriptomic features. This more easily allows collaborative projects across multiple sites to acquire skin biopsy specimens in a uniform method for single-cell sequencing to identify dominant cell types and pathways of disease. This is applicable to a multitude of skin disorders, including autoimmune conditions with cutaneous pathology like localized scleroderma, systemic sclerosis, cutaneous lupus erythematosus, and dermatomyositis. Applying single-cell RNA sequencing of the skin to large numbers of patients will afford new opportunities to discover autoimmune disease biomarkers, targets for therapeutic drug development, and the molecular stratification of disease.

## Supplementary information

**Additional file 1 : Supplemental Figure 1**. Quality control (QC) metrics of single cell data between cryopreserved (Cryostor CS10®, pink) and fresh (RPMI, blue) samples before filtering techniques are applied. QC metrics, including A) the number of unique genes, B) the number of total molecules, and C) the percentage of reads that map to the mitochondrial genome, all demonstrate equivalence between preservation methods before filtering and normalization with D) patient representation between clusters from three patients demonstrated (P1 – SC222, SC223, P2 – SC267, SC268 and P3 – SC272, SC273; Cryostor and RPMI samples respectively). **Supplemental Figure 2**. Heat map of single cell data clustering of combined cryopreserved (Cryostor CS10®) and fresh (RPMI) samples after filtering techniques are applied. Graph shows the top 5 expressed genes per the 9 identified cell groupings in the dataset. **Supplemental Figure 3.** Without filtering methods, samples maintain even disbursement with clustering via t-Distributed stochastic neighbor embedding (t-SNE). A) Three patients overlap well with cellular transcriptomic expression across the cell clusters. B) Cryostor**®** (frozen) and RPMI (fresh) preservation methods show even dispersion across cell clusters. C) Individual patient with paired frozen and fresh specimens demonstrate even dispersion. These t-SNE plots represent 15,910 skin cells, derived from 3 patients with LS (3 fresh and 3 cryopreserved samples with 9245 and 6665 cells respectively). **Supplemental Figure 4**. Correlation of average genetic expression for major cell groups shows high correlation between sample types. Fresh and cryopreserved samples correlated significantly within cell groups including keratinocytes, T/NK cells, DC/macrophages, fibroblasts, and pericytes even without filtering and normalization. Each point on the correlation plots display the average UMI counts for each gene across all cells for each major cell group. **Supplemental Figure 5**. Gene expression profiling of known keratinocyte sub clusters from He et al. 2020 were used to define cell clusters. Subclustering of keratinocytes revealed 12 distinct groups of cells within this group which were further identified using defined gene signatures. Gene signatures are presented via feature plot. **Supplemental Figure 6**. t-Distributed stochastic neighbor embedding plot for 4252 keratinocytes, derived from 3 patients with LS (3 fresh and 3 cryopreserved samples with 3254 and 998 cells respectively). After normalization, tSNE plots show relatively even dispersion of different processing type in each cluster given the much larger overall number of fresh keratinocytes compared to cryopreserved. Bottom separated by patient. **Supplemental Table 1.** Transcriptomic expression of genes within cell types were similar between preservation methods in frozen media (Cryostor® CS10) compared to fresh media (RPMI). **Supplemental Table 2**. Wilcoxon ranked statistical testing between Cryostor® and fresh cell numbers demonstrated no significant difference between preservation method. **Supplemental Table 3**. Differentially expressed genes between Cryostor® and fresh skin samples.

## Data Availability

In addition to the data included in the manuscript and the supplementary files, additional datasets analyzed during the current study are available from the corresponding author on reasonable request. RNA single-cell sequencing data generated from the study is deposited on NCBI Gene Expression Omnibus (GSE160536).

## Abbreviations

AMP: Accelerating Medicines Partnership RA/SLE Network
BSA: Bovine serum albumin
DC: Dendritic cells
DMSO: Dimethylsulfoxide
FLG: Filaggrin
GEMs: Gel Bead-In-Emulsions
GO: Gene Ontology Enrichment Analysis
GSEA®: Gene Set Enrichment Analysis
IFN-y: Interferon gamma
LS: Localized scleroderma
MAC: Morphea in Adults and Children
NRCOS: National Registry of Childhood Onset Scleroderma
NK: Natural killer
PBMC: Peripheral blood mononuclear cells
PBS: Phosphate-buffered saline
PCA: Principal component analysis
ScRNA: Single-cell RNA sequencing
SLM: Smart local moving algorithm
t-SNE: t-Distributed stochastic neighbor embedding
UMI: Unique molecular identifier
