# Correction to: Single-cell transcriptome conservation in a comparative analysis of fresh and cryopreserved human skin tissue: pilot in localized scleroderma

**DOI:** 10.1186/s13075-021-02490-2

**Published:** 2021-04-06

**Authors:** Emily Mirizio, Tracy Tabib, Xinjun Wang, Wei Chen, Christopher Liu, Robert Lafyatis, Heidi Jacobe, Kathryn S. Torok

**Affiliations:** 1 Division of Rheumatology, Department of Pediatrics, Children’s Hospital of Pittsburgh, University of Pittsburgh, Pittsburgh, PA USA; 2 Division of Rheumatology, Department of Medicine, University of Pittsburgh, Pittsburgh, PA USA; 3 Division of Pediatric Pulmonary Medicine, UPMC Children’s Hospital of Pittsburgh, University of Pittsburgh, Pittsburgh, PA USA; 4 Department of Dermatology, University of Texas Southwestern Medical Center, Dallas, TX USA; 5 University of Pittsburgh Medical Center Children’s Hospital of Pittsburgh Faculty Pavilion, 3rd floor, Office 3117 4401 Penn Avenue, Pittsburgh, PA 15237 USA

**Correction to: Arthritis Res Ther (2020) 22:263**

**https://doi.org/10.1186/s13075-020-02343-4**

Following publication of the original article [1], the authors reported an error in one of the author’s name. The 3rd author should be **Xinjun Wang**, as this was incorrectly given as Xiao Wang by the corresponding author during proofing stage.

The original article has been corrected.
