# Supplementary material for: Single-cell transcriptome conservation in a comparative analysis of fresh and cryopreserved human skin tissue: pilot in localized scleroderma
Source: Arthritis Res Ther. 2020 Nov 9;22:263. doi: 10.1186/s13075-020-02343-4 (PMC7654179; doi:10.1186/s13075-020-02343-4)
Supplement: Supplementary file 1 — Additional file 1 : Supplemental Figure 1. Quality control (QC) metrics of single cell data between cryopreserved (Cryostor CS10®, pink) and fresh (RPMI, blue) samples before filtering techniques are applied. QC metrics, including A) the number of unique genes, B) the number of total molecules, and C) the percentage of reads that map to the mitochondrial genome, all demonstrate equivalence between preservation methods before filtering and normalization with D) patient representation between clusters from three patients demonstrated (P1 – SC222, SC223, P2 – SC267, SC268 and P3 – SC272, SC273; Cryostor and RPMI samples respectively). Supplemental Figure 2. Heat map of single cell data clustering of combined cryopreserved (Cryostor CS10®) and fresh (RPMI) samples after filtering techniques are applied. Graph shows the top 5 expressed genes per the 9 identified cell groupings in the dataset. Supplemental Figure 3. Without filtering methods, samples maintain even disbursement with clustering via t-Distributed stochastic neighbor embedding (t-SNE). A) Three patients overlap well with cellular transcriptomic expression across the cell clusters. B) Cryostor® (frozen) and RPMI (fresh) preservation methods show even dispersion across cell clusters. C) Individual patient with paired frozen and fresh specimens demonstrate even dispersion. These t-SNE plots represent 15,910 skin cells, derived from 3 patients with LS (3 fresh and 3 cryopreserved samples with 9245 and 6665 cells respectively). Supplemental Figure 4. Correlation of average genetic expression for major cell groups shows high correlation between sample types. Fresh and cryopreserved samples correlated significantly within cell groups including keratinocytes, T/NK cells, DC/macrophages, fibroblasts, and pericytes even without filtering and normalization. Each point on the correlation plots display the average UMI counts for each gene across all cells for each major cell group. Supplemental Figure 5. Gene expression p [file 13075_2020_2343_MOESM1_ESM.zip › Final_Supp_whitepaper.docx]

**SUPPLEMENT**

**Pre-filtered Data Analysis:**

To determine if normalization techniques influenced clustering and subsequent sample analysis, raw cell data was clustered using a smart local moving algorithm (SLM) (no filtering based on overall or mitochondrial gene expression and no regression using the Seruat function SCTransform). Cell populations were identified based on gene markers in the associated transcriptomes and visualized by t-distributed stochastic neighbor embedding (t-SNE).^35^ As seen in **Supplemental Figures 1 and** **2**, the number of feature genes, RNA, and mitochondrial DNA was also analogous between sample types (Cryostor® and RPMI) for each individual patient sample and collectively.

The average expression of genes for major cell groups, such as keratinocytes, T/NK cells, DC/macrophages, fibroblasts, and pericytes, demonstrated strong correlation even without filtering and normalization (**Supplemental Figure 2, Supplemental Table 1**). Spearman’s rho correlations are still strong but lower for the unfiltered data compared to the filtered and normalized data.

**Supplemental Table 1.** Transcriptomic expression of genes within cell types were similar between preservation methods in frozen media (Cryostor® CS10) compared to fresh media (RPMI).

|  | Spearman’s Rho | p-value |
| --- | --- | --- |
| T cells | 0.90 | < 0.0001 |
| Macrophages | 0.87 | < 0.0001 |
| Dendritic Cells | 0.87 | < 0.0001 |
| Keratinocytes | 0.90 | < 0.0001 |
| Fibroblasts | 0.68 | < 0.0001 |
| Pericytes | 0.94 | < 0.0001 |

**Post-Normalization of Data:**

**Supplemental Table 2**. Wilcoxon ranked statistical testing between Cryostor® and fresh cell numbers demonstrated no significant difference between preservation method.

|  | p-value |
| --- | --- |
| T cells | 0.5000 |
| Macrophages | >0.9999 |
| Dendritic Cells | 0.5000 |
| Keratinocytes | 0.2500 |
| Fibroblasts | 0.7500 |
| Pericytes | 0.7500 |

**Supplemental Table 3**. Differentially expressed genes between Cryostor® and fresh skin samples.

|  | **P Value** | **Average log Fold Change** | **% of Cells in PCA 1** | **% of Cells in PCA 2** | **Adjusted P Value** |
| --- | --- | --- | --- | --- | --- |
| HBB | 0.00E+00 | -1.520989 | 0.011 | 0.386 | 0.00 |
| KRT10 | 0.00E+00 | -1.3492388 | 0.449 | 0.752 | 0.00 |
| KRT1 | 0.00E+00 | -1.1858538 | 0.209 | 0.507 | 0.00 |
| DMKN | 0.00E+00 | -1.1793984 | 0.183 | 0.497 | 0.00 |
| KRT2 | 3.78E-253 | -1.1113332 | 0.017 | 0.205 | < 0.0001 |
| KRTDAP | 8.76E-306 | -1.0710353 | 0.053 | 0.301 | < 0.0001 |
| PERP | 2.89E-280 | -0.9871607 | 0.401 | 0.636 | < 0.0001 |
| MT2A | 3.80E-111 | 0.9831548 | 0.779 | 0.696 | < 0.0001 |
| SFN | 0.00E+00 | -0.9807685 | 0.285 | 0.581 | < 0.0001 |
| LGALS7B | 6.03E-308 | -0.9807484 | 0.078 | 0.335 | < 0.0001 |
| KRT14 | 6.98E-214 | -0.9510459 | 0.31 | 0.562 | < 0.0001 |
| DSP | 5.67E-270 | -0.8763529 | 0.13 | 0.38 | < 0.0001 |
| COL1A2 | 1.22E-07 | -0.8196736 | 0.135 | 0.159 | < 0.0001 |
| AQP3 | 6.70E-267 | -0.8171361 | 0.145 | 0.387 | < 0.0001 |
| LYPD3 | 2.41E-242 | -0.8063044 | 0.075 | 0.289 | < 0.0001 |
| COL1A1 | 6.34E-09 | -0.7832211 | 0.141 | 0.169 | < 0.0001 |
| COL3A1 | 7.17E-13 | -0.7447627 | 0.108 | 0.142 | < 0.0001 |
| LY6D | 5.76E-267 | -0.7058591 | 0.08 | 0.319 | < 0.0001 |
| KRT5 | 2.02E-114 | -0.7031441 | 0.129 | 0.284 | < 0.0001 |
| TM4SF1 | 1.07E-97 | 0.6952887 | 0.361 | 0.207 | < 0.0001 |
| HBA2 | 0.00E+00 | -0.6888931 | 0.005 | 0.223 | < 0.0001 |
| S100A14 | 3.18E-282 | -0.687875 | 0.086 | 0.33 | < 0.0001 |
| CCL2 | 1.96E-14 | 0.6630767 | 0.387 | 0.336 | < 0.0001 |
| SERPINB2 | 3.56E-268 | -0.585732 | 0.026 | 0.23 | < 0.0001 |
| RGS16 | 1.72E-36 | 0.5786785 | 0.27 | 0.189 | < 0.0001 |
| FTH1 | 1.27E-159 | 0.5688112 | 0.991 | 0.93 | < 0.0001 |
| CXCL14 | 8.47E-242 | -0.5604027 | 0.201 | 0.456 | < 0.0001 |
| SOCS3 | 3.08E-37 | 0.5443241 | 0.578 | 0.535 | < 0.0001 |
| IL6 | 4.40E-69 | 0.5294884 | 0.171 | 0.077 | < 0.0001 |
| DSC3 | 6.21E-289 | -0.524379 | 0.059 | 0.294 | < 0.0001 |
| CXCL2 | 1.22E-74 | 0.5164706 | 0.242 | 0.129 | < 0.0001 |
| SBSN | 6.47E-206 | -0.5123408 | 0.017 | 0.177 | < 0.0001 |
| SOD2 | 2.30E-45 | 0.4981968 | 0.454 | 0.375 | < 0.0001 |
| ZFP36 | 5.90E-25 | 0.4901233 | 0.834 | 0.83 | < 0.0001 |
| KLF5 | 6.30E-214 | -0.4807839 | 0.114 | 0.321 | < 0.0001 |
| SERPINB5 | 4.88E-259 | -0.4504552 | 0.054 | 0.272 | < 0.0001 |
| RGCC | 3.48E-76 | 0.4492679 | 0.324 | 0.205 | < 0.0001 |
| DST | 1.55E-114 | -0.4427954 | 0.124 | 0.271 | < 0.0001 |
| DCN | 7.84E-51 | -0.4388673 | 0.099 | 0.19 | < 0.0001 |
| S100A2 | 5.35E-83 | -0.4382949 | 0.073 | 0.181 | < 0.0001 |
| CCL27 | 5.35E-210 | -0.4354395 | 0.076 | 0.285 | < 0.0001 |
| CD74 | 2.76E-178 | 0.4349829 | 0.806 | 0.591 | < 0.0001 |
| TMSB10 | 3.84E-209 | 0.432764 | 0.968 | 0.886 | < 0.0001 |
| CXCL3 | 6.28E-42 | 0.4307595 | 0.171 | 0.097 | < 0.0001 |
| GEM | 1.27E-20 | 0.4248469 | 0.178 | 0.131 | < 0.0001 |
| SLC38A2 | 4.81E-205 | -0.4237742 | 0.313 | 0.535 | < 0.0001 |
| PKP1 | 4.16E-248 | -0.4229411 | 0.041 | 0.243 | < 0.0001 |
| TMSB4X | 4.67E-140 | 0.4220018 | 0.992 | 0.992 | < 0.0001 |
| TACSTD2 | 4.56E-176 | -0.4204307 | 0.159 | 0.368 | < 0.0001 |
| G0S2 | 2.91E-53 | 0.4200443 | 0.167 | 0.083 | < 0.0001 |
| LMNA | 9.64E-74 | 0.413733 | 0.873 | 0.852 | < 0.0001 |
| DSG1 | 4.23E-242 | -0.4034753 | 0.028 | 0.216 | < 0.0001 |
| LGALS7 | 1.20E-190 | -0.394968 | 0.055 | 0.235 | < 0.0001 |
| COL6A2 | 6.55E-03 | -0.3864215 | 0.181 | 0.193 | < 0.0001 |
| HES1 | 1.40E-02 | 0.3839771 | 0.478 | 0.524 | < 0.0001 |
| HSP90AB1 | 1.11E-159 | -0.3811325 | 0.88 | 0.941 | < 0.0001 |
| MT1E | 2.17E-07 | 0.3768156 | 0.278 | 0.256 | < 0.0001 |
| SRGN | 4.91E-64 | 0.369616 | 0.412 | 0.286 | < 0.0001 |
| CRIP1 | 8.88E-58 | 0.3685213 | 0.506 | 0.42 | < 0.0001 |
| ISG15 | 1.30E-94 | 0.3680795 | 0.303 | 0.166 | < 0.0001 |
| MT1A | 3.97E-64 | 0.3653374 | 0.101 | 0.035 | < 0.0001 |
| FOS | 2.40E-02 | 0.3641559 | 0.7 | 0.766 | < 0.0001 |
| PHLDA1 | 5.19E-98 | 0.3637548 | 0.341 | 0.198 | < 0.0001 |
| C11orf96 | 7.90E-43 | 0.3628384 | 0.153 | 0.084 | < 0.0001 |
| HLA-DPB1 | 2.37E-96 | 0.3584742 | 0.544 | 0.387 | < 0.0001 |
| INSIG1 | 1.70E-90 | 0.354984 | 0.322 | 0.184 | < 0.0001 |
| FOSB | 4.36E-32 | 0.3455425 | 0.42 | 0.356 | < 0.0001 |
| JUND | 2.26E-166 | 0.3423268 | 0.437 | 0.239 | < 0.0001 |
| IFITM1 | 3.29E-113 | 0.340745 | 0.37 | 0.212 | < 0.0001 |
| CEBPD | 6.64E-02 | 0.3391112 | 0.552 | 0.587 | < 0.0001 |
| SAT1 | 5.91E-63 | 0.3375857 | 0.784 | 0.711 | < 0.0001 |
| DEGS1 | 2.04E-160 | -0.3333961 | 0.152 | 0.337 | < 0.0001 |
| ICAM1 | 4.20E-79 | 0.3311081 | 0.279 | 0.161 | < 0.0001 |
| RPLP1 | 3.46E-107 | -0.3298414 | 0.999 | 1 | < 0.0001 |
| AREG | 1.68E-75 | -0.3295697 | 0.088 | 0.195 | < 0.0001 |
| TMEM45A | 5.97E-186 | -0.3285456 | 0.052 | 0.224 | < 0.0001 |
| AC020916.1 | 8.03E-73 | 0.3260349 | 0.272 | 0.159 | < 0.0001 |
| POSTN | 1.86E-63 | -0.3251117 | 0.047 | 0.127 | < 0.0001 |
| HLA-DRA | 3.74E-114 | 0.3228345 | 0.661 | 0.48 | < 0.0001 |
| KLF2 | 3.55E-91 | 0.3175366 | 0.318 | 0.184 | < 0.0001 |
| IFI27 | 1.73E-46 | 0.3141403 | 0.522 | 0.424 | < 0.0001 |
| GPNMB | 3.29E-177 | -0.3121533 | 0.156 | 0.364 | < 0.0001 |
| S100A4 | 4.34E-47 | 0.3085241 | 0.709 | 0.619 | < 0.0001 |
| MT-CO1 | 2.60E-22 | 0.3032182 | 0.994 | 0.986 | < 0.0001 |
| MT-ND4L | 6.99E-44 | -0.2989058 | 0.49 | 0.585 | < 0.0001 |
| COL6A1 | 8.78E-03 | -0.2971583 | 0.147 | 0.158 | < 0.0001 |
| CXADR | 3.72E-210 | -0.2958577 | 0.05 | 0.237 | < 0.0001 |
| SLC2A3 | 3.22E-108 | 0.2942209 | 0.441 | 0.269 | < 0.0001 |
| B2M | 2.08E-166 | 0.2930444 | 0.999 | 1 | < 0.0001 |
| MGP | 5.26E-44 | 0.2902957 | 0.337 | 0.232 | < 0.0001 |
| HLA-B | 9.21E-137 | 0.288997 | 0.976 | 0.969 | < 0.0001 |
| TUBB2A | 4.88E-114 | -0.2880751 | 0.193 | 0.354 | < 0.0001 |
| CCL5 | 1.07E-35 | 0.284514 | 0.127 | 0.067 | < 0.0001 |
| DUSP1 | 2.40E-01 | 0.2820402 | 0.742 | 0.783 | < 0.0001 |
| ACKR1 | 1.28E-46 | 0.2806001 | 0.209 | 0.12 | < 0.0001 |
| MT-CO3 | 4.16E-11 | 0.2800303 | 0.997 | 0.989 | < 0.0001 |
| ATF3 | 2.47E-01 | 0.2779008 | 0.427 | 0.464 | < 0.0001 |
| JUN | 3.72E-11 | 0.2755736 | 0.707 | 0.808 | < 0.0001 |
| JUNB | 5.29E-01 | 0.2753514 | 0.873 | 0.91 | < 0.0001 |
| MYC | 1.34E-68 | -0.2745936 | 0.421 | 0.546 | < 0.0001 |
| S100A6 | 3.77E-119 | 0.2744757 | 0.942 | 0.79 | < 0.0001 |
| TRIM29 | 9.47E-184 | -0.2743827 | 0.058 | 0.235 | < 0.0001 |
| KRT17 | 3.25E-127 | -0.271258 | 0.037 | 0.164 | < 0.0001 |
| HLA-DRB1 | 1.13E-92 | 0.2710589 | 0.648 | 0.486 | < 0.0001 |
| HLA-DPA1 | 1.85E-69 | 0.2705426 | 0.465 | 0.326 | < 0.0001 |
| GJA1 | 1.99E-110 | -0.2685899 | 0.11 | 0.254 | < 0.0001 |
| APOE | 1.98E-94 | -0.2672471 | 0.202 | 0.36 | < 0.0001 |
| C2CD4B | 2.99E-55 | 0.2671919 | 0.107 | 0.042 | < 0.0001 |
| CXCR4 | 3.43E-48 | 0.2668054 | 0.271 | 0.172 | < 0.0001 |
| GADD45B | 4.32E-15 | 0.2662719 | 0.647 | 0.607 | < 0.0001 |
| IRF6 | 3.20E-201 | -0.2654341 | 0.043 | 0.219 | < 0.0001 |
| AHNAK2 | 8.40E-180 | -0.2646659 | 0.042 | 0.204 | < 0.0001 |
| CD52 | 2.23E-43 | 0.2639401 | 0.231 | 0.144 | < 0.0001 |
| PLK2 | 1.97E-102 | -0.2588871 | 0.163 | 0.311 | < 0.0001 |
| TSC22D1 | 6.24E-15 | 0.2587909 | 0.366 | 0.32 | < 0.0001 |
| IFITM3 | 2.29E-61 | 0.2561559 | 0.704 | 0.602 | < 0.0001 |
| RSRP1 | 5.26E-96 | -0.2559912 | 0.395 | 0.542 | < 0.0001 |
| CKS2 | 3.97E-69 | -0.2552016 | 0.208 | 0.326 | < 0.0001 |
| DNAJA1 | 2.58E-55 | -0.2545255 | 0.655 | 0.724 | < 0.0001 |
| SDCBP | 1.49E-86 | 0.2529926 | 0.521 | 0.377 | < 0.0001 |
| ANXA2 | 1.05E-98 | -0.2511617 | 0.81 | 0.865 | < 0.0001 |
| DUSP2 | 2.48E-22 | 0.2509386 | 0.257 | 0.198 | < 0.0001 |
| CDKN1A | 1.53E-08 | 0.250611 | 0.584 | 0.599 | < 0.0001 |

**Keratinocyte Comparison:**

He et al. identified 7 sub clusters of keratinocytes in their skin samples with a major decrease in the stratum corneum cells expressing late differentiation markers (FLG) in cryopreserved sample compared to fresh^37^. Using a similar clustering method for the three patient samples from our combined cohorts found similar sub clusters. Sub clustering analysis of keratinocytes, using an unsupervised graph-based clustering algorithm, of 4,252 cells from 3 patients with 1 fresh and 1 frozen sample each (6 total samples) identified thirteen distinct clusters of cells displayed by color on the t-Distributed Stochastic Neighbor Embedding (t-SNE) plot in **Supplemental Figure 4**. Briefly, basal keratinocytes were identified by cell expression of KRT5, KRT14, and KRT15; suprabasal keratinocytes by KRT1, KRT10, KRT6A, KRT16, S100A7, and S100A8; proliferating keratinocytes by UBE2C and TOP2A; combined subpopulation of IRS and sebaceous gland cells (IRS-sebaceous) by KRT79, FASN, THRSP, ELOVL5, FADS2, ACSBG1 , and APOC1; outer root sheath cells by KRT6B and KRT17; “Channel-ATPase” keratinocytes by ATP1B1, ATP1A1, DEFB1 and SAT1; “channel-gap” keratinocytes by GJB2 and GJA1. These sub clusters were equally represented in our data sets (**Supplemental Figure 4**). Due to overall higher numbers of fresh keratinocytes, cryopreserved samples were underrepresented in sub cluster of keratinocytes (**Supplemental Figure 5**). In line with He et al’s findings, late differentiation marker FLG was not expressed from cryopreserved samples.

**Figure Legends:**

**Supplemental Figure 1: Quality control (QC) metrics of single cell data between cryopreserved (Cryostor CS10®, pink) and fresh (RPMI, blue) samples before filtering techniques are applied.** QC metrics, including A) the number of unique genes, B) the number of total molecules, and C) the percentage of reads that map to the mitochondrial genome, all demonstrate equivalence between preservation methods before filtering and normalization with D) patient representation between clusters from three patients demonstrated (P1 – SC222, SC223, P2 – SC267, SC268 and P3 – SC272, SC273; Cryostor and RPMI samples respectively).

**Supplemental Figure 2: Heat map of single cell data clustering of combined cryopreserved (Cryostor CS10®) and fresh (RPMI) samples after filtering techniques are applied.** Graph shows the top 5 expressed genes per the 9 identified cell groupings in the dataset.

**Supplemental Figure 3: Without filtering methods, samples maintain even disbursement with clustering via t-Distributed stochastic neighbor embedding (t-SNE).** A) Three patients overlap well with cellular transcriptomic expression across the cell clusters. B) Cryostor **®** (frozen) and RPMI (fresh) preservation methods show even dispersion across cell clusters. C) Individual patient with paired frozen and fresh specimens demonstrate even dispersion. These t-SNE plots represent 15,910 skin cells, derived from 3 patients with LS (3 fresh and 3 cryopreserved samples with 9,245 and 6,665 cells respectively).

**Supplemental Figure 4:** **Correlation of average genetic expression for major cell groups shows high correlation between sample types**. Fresh and cryopreserved samples correlated significantly within cell groups including keratinocytes, T/NK cells, DC/macrophages, fibroblasts, and pericytes even without filtering and normalization. Each point on the correlation plots display the average UMI counts for each gene across all cells for each major cell group.

**Supplemental Figure 5: Gene expression profiling of known keratinocyte sub clusters from He et al 2020 were used to define cell clusters.** Subclustering of keratinocytes revealed 12 distinct groups of cells within this group which were further identified using defined gene signatures. Gene signatures are presented via feature plot.

**Supplemental Figure 6: t-Distributed stochastic neighbor embedding plot for 4,252 keratinocytes, derived from 3 patients with LS (3 fresh and 3 cryopreserved samples with 3,254 and 998 cells respectively).** After normalization, tSNE plots show relatively even dispersion of different processing type in each cluster given the much larger overall number of fresh keratinocytes compared to cryopreserved. Bottom separated by patient.
