# Supplementary figures and images for: Single-cell transcriptome conservation in a comparative analysis of fresh and cryopreserved human skin tissue: pilot in localized scleroderma
Source: Arthritis Res Ther. 2020 Nov 9;22:263. doi: 10.1186/s13075-020-02343-4 (PMC7654179; doi:10.1186/s13075-020-02343-4)

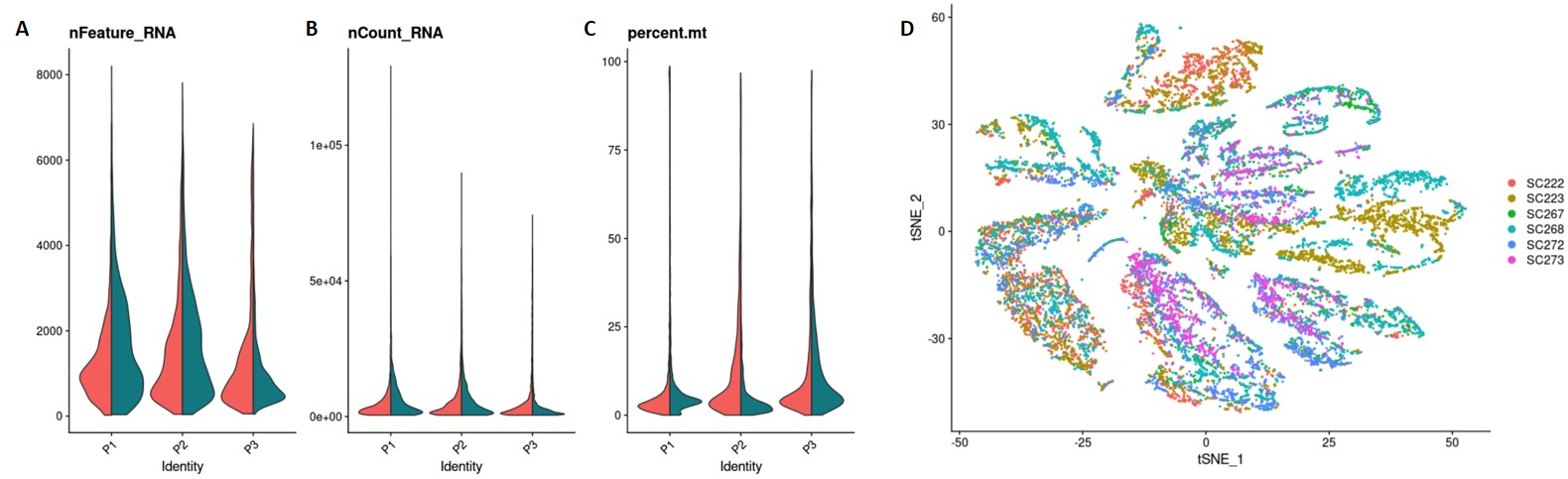

Supplement: Supplementary file 1 — Additional file 1 : Supplemental Figure 1. Quality control (QC) metrics of single cell data between cryopreserved (Cryostor CS10®, pink) and fresh (RPMI, blue) samples before filtering techniques are applied. QC metrics, including A) the number of unique genes, B) the number of total molecules, and C) the percentage of reads that map to the mitochondrial genome, all demonstrate equivalence between preservation methods before filtering and normalization with D) patient representation between clusters from three patients demonstrated (P1 – SC222, SC223, P2 – SC267, SC268 and P3 – SC272, SC273; Cryostor and RPMI samples respectively). Supplemental Figure 2. Heat map of single cell data clustering of combined cryopreserved (Cryostor CS10®) and fresh (RPMI) samples after filtering techniques are applied. Graph shows the top 5 expressed genes per the 9 identified cell groupings in the dataset. Supplemental Figure 3. Without filtering methods, samples maintain even disbursement with clustering via t-Distributed stochastic neighbor embedding (t-SNE). A) Three patients overlap well with cellular transcriptomic expression across the cell clusters. B) Cryostor® (frozen) and RPMI (fresh) preservation methods show even dispersion across cell clusters. C) Individual patient with paired frozen and fresh specimens demonstrate even dispersion. These t-SNE plots represent 15,910 skin cells, derived from 3 patients with LS (3 fresh and 3 cryopreserved samples with 9245 and 6665 cells respectively). Supplemental Figure 4. Correlation of average genetic expression for major cell groups shows high correlation between sample types. Fresh and cryopreserved samples correlated significantly within cell groups including keratinocytes, T/NK cells, DC/macrophages, fibroblasts, and pericytes even without filtering and normalization. Each point on the correlation plots display the average UMI counts for each gene across all cells for each major cell group. Supplemental Figure 5. Gene expression p [file 13075_2020_2343_MOESM1_ESM.zip › Supp. Figure 1.jpg]

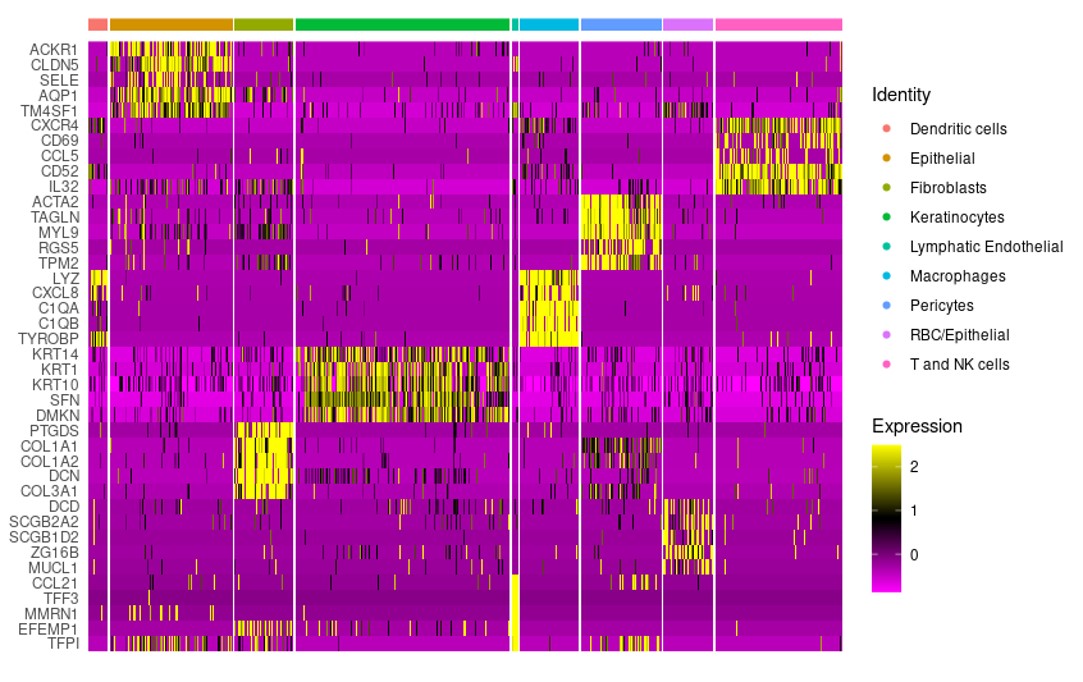

Supplement: Supplementary file 1 — Additional file 1 : Supplemental Figure 1. Quality control (QC) metrics of single cell data between cryopreserved (Cryostor CS10®, pink) and fresh (RPMI, blue) samples before filtering techniques are applied. QC metrics, including A) the number of unique genes, B) the number of total molecules, and C) the percentage of reads that map to the mitochondrial genome, all demonstrate equivalence between preservation methods before filtering and normalization with D) patient representation between clusters from three patients demonstrated (P1 – SC222, SC223, P2 – SC267, SC268 and P3 – SC272, SC273; Cryostor and RPMI samples respectively). Supplemental Figure 2. Heat map of single cell data clustering of combined cryopreserved (Cryostor CS10®) and fresh (RPMI) samples after filtering techniques are applied. Graph shows the top 5 expressed genes per the 9 identified cell groupings in the dataset. Supplemental Figure 3. Without filtering methods, samples maintain even disbursement with clustering via t-Distributed stochastic neighbor embedding (t-SNE). A) Three patients overlap well with cellular transcriptomic expression across the cell clusters. B) Cryostor® (frozen) and RPMI (fresh) preservation methods show even dispersion across cell clusters. C) Individual patient with paired frozen and fresh specimens demonstrate even dispersion. These t-SNE plots represent 15,910 skin cells, derived from 3 patients with LS (3 fresh and 3 cryopreserved samples with 9245 and 6665 cells respectively). Supplemental Figure 4. Correlation of average genetic expression for major cell groups shows high correlation between sample types. Fresh and cryopreserved samples correlated significantly within cell groups including keratinocytes, T/NK cells, DC/macrophages, fibroblasts, and pericytes even without filtering and normalization. Each point on the correlation plots display the average UMI counts for each gene across all cells for each major cell group. Supplemental Figure 5. Gene expression p [file 13075_2020_2343_MOESM1_ESM.zip › Supp. Figure 2.jpg]

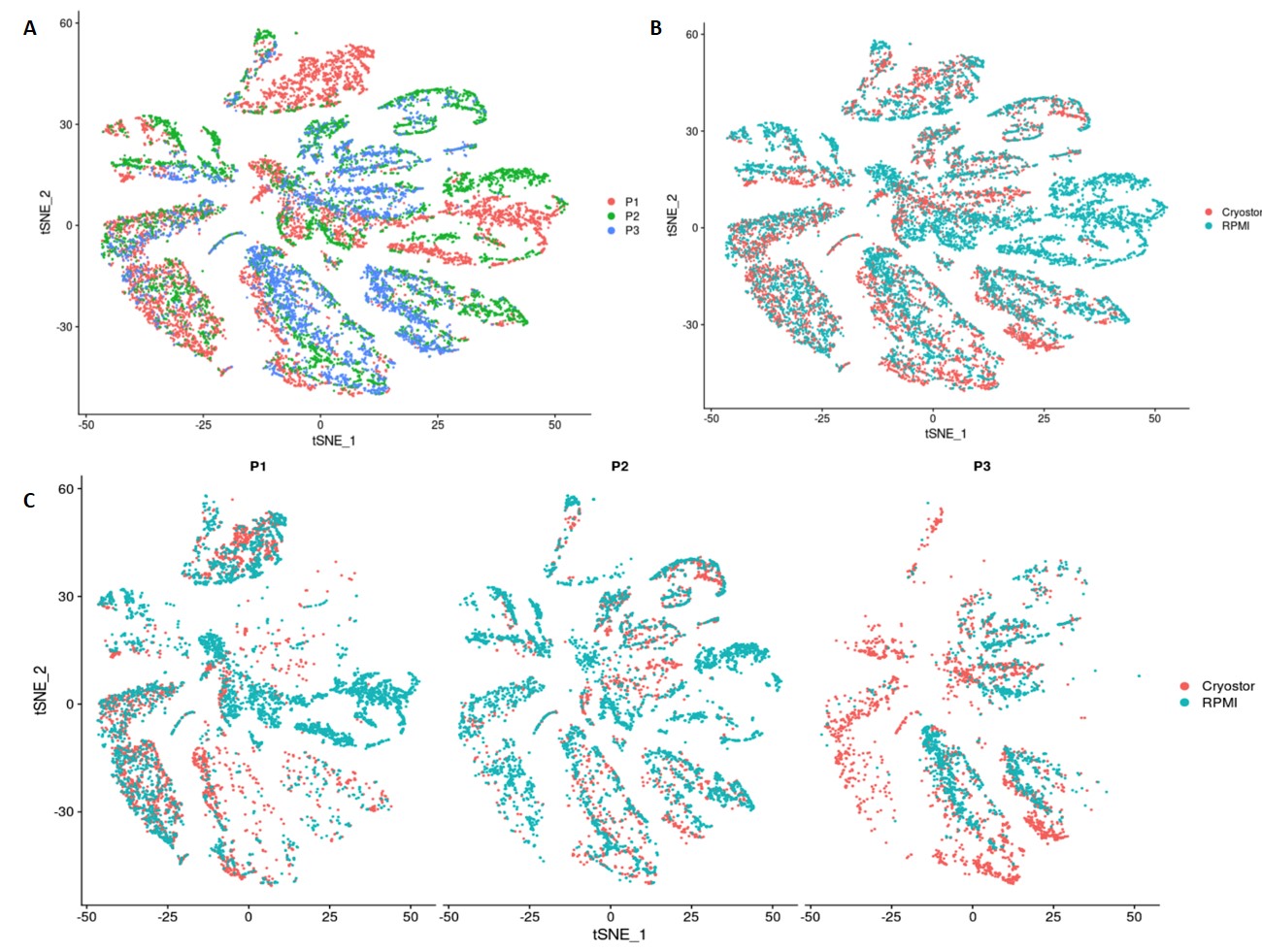

Supplement: Supplementary file 1 — Additional file 1 : Supplemental Figure 1. Quality control (QC) metrics of single cell data between cryopreserved (Cryostor CS10®, pink) and fresh (RPMI, blue) samples before filtering techniques are applied. QC metrics, including A) the number of unique genes, B) the number of total molecules, and C) the percentage of reads that map to the mitochondrial genome, all demonstrate equivalence between preservation methods before filtering and normalization with D) patient representation between clusters from three patients demonstrated (P1 – SC222, SC223, P2 – SC267, SC268 and P3 – SC272, SC273; Cryostor and RPMI samples respectively). Supplemental Figure 2. Heat map of single cell data clustering of combined cryopreserved (Cryostor CS10®) and fresh (RPMI) samples after filtering techniques are applied. Graph shows the top 5 expressed genes per the 9 identified cell groupings in the dataset. Supplemental Figure 3. Without filtering methods, samples maintain even disbursement with clustering via t-Distributed stochastic neighbor embedding (t-SNE). A) Three patients overlap well with cellular transcriptomic expression across the cell clusters. B) Cryostor® (frozen) and RPMI (fresh) preservation methods show even dispersion across cell clusters. C) Individual patient with paired frozen and fresh specimens demonstrate even dispersion. These t-SNE plots represent 15,910 skin cells, derived from 3 patients with LS (3 fresh and 3 cryopreserved samples with 9245 and 6665 cells respectively). Supplemental Figure 4. Correlation of average genetic expression for major cell groups shows high correlation between sample types. Fresh and cryopreserved samples correlated significantly within cell groups including keratinocytes, T/NK cells, DC/macrophages, fibroblasts, and pericytes even without filtering and normalization. Each point on the correlation plots display the average UMI counts for each gene across all cells for each major cell group. Supplemental Figure 5. Gene expression p [file 13075_2020_2343_MOESM1_ESM.zip › Supp. Figure 3.jpg]

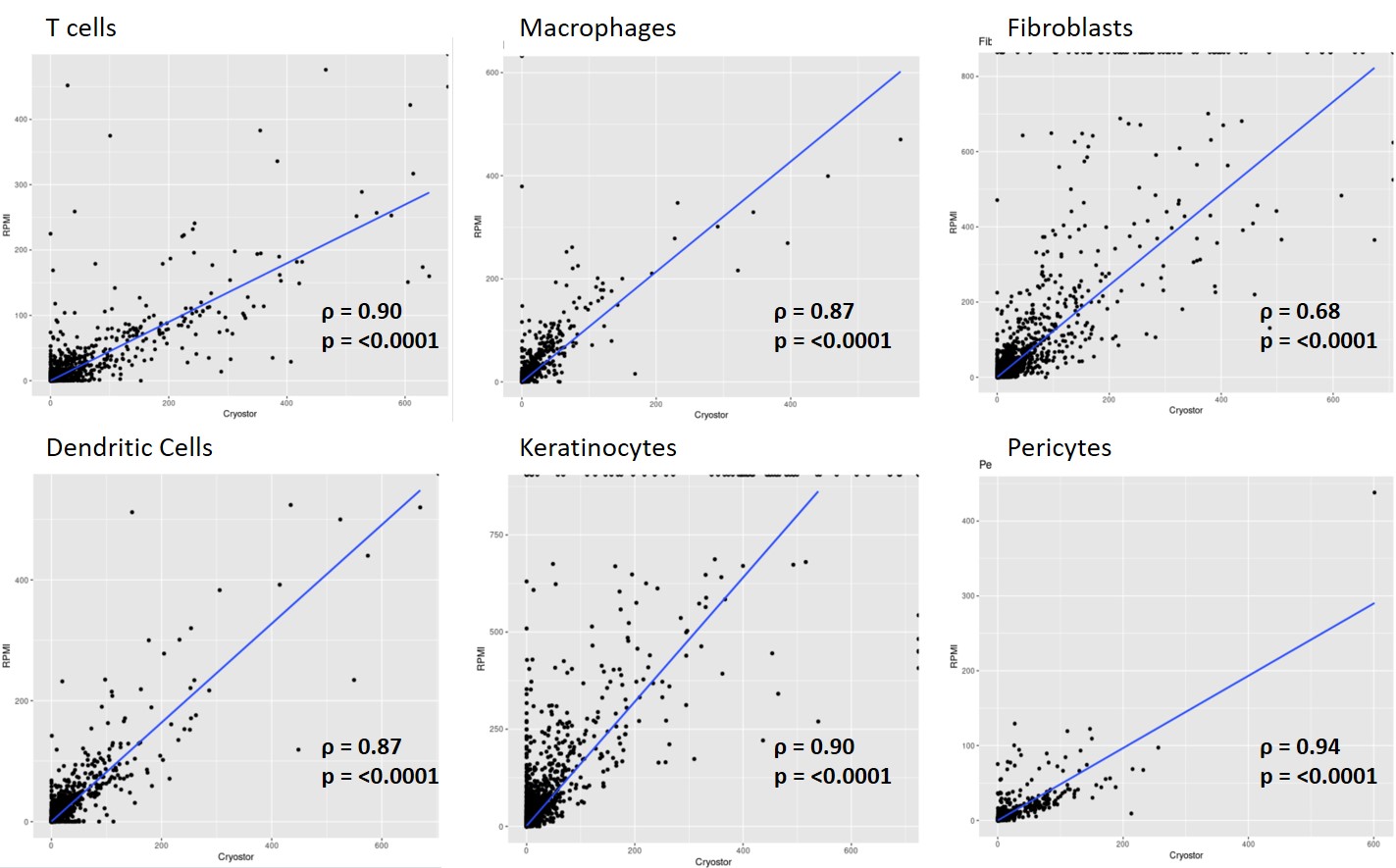

Supplement: Supplementary file 1 — Additional file 1 : Supplemental Figure 1. Quality control (QC) metrics of single cell data between cryopreserved (Cryostor CS10®, pink) and fresh (RPMI, blue) samples before filtering techniques are applied. QC metrics, including A) the number of unique genes, B) the number of total molecules, and C) the percentage of reads that map to the mitochondrial genome, all demonstrate equivalence between preservation methods before filtering and normalization with D) patient representation between clusters from three patients demonstrated (P1 – SC222, SC223, P2 – SC267, SC268 and P3 – SC272, SC273; Cryostor and RPMI samples respectively). Supplemental Figure 2. Heat map of single cell data clustering of combined cryopreserved (Cryostor CS10®) and fresh (RPMI) samples after filtering techniques are applied. Graph shows the top 5 expressed genes per the 9 identified cell groupings in the dataset. Supplemental Figure 3. Without filtering methods, samples maintain even disbursement with clustering via t-Distributed stochastic neighbor embedding (t-SNE). A) Three patients overlap well with cellular transcriptomic expression across the cell clusters. B) Cryostor® (frozen) and RPMI (fresh) preservation methods show even dispersion across cell clusters. C) Individual patient with paired frozen and fresh specimens demonstrate even dispersion. These t-SNE plots represent 15,910 skin cells, derived from 3 patients with LS (3 fresh and 3 cryopreserved samples with 9245 and 6665 cells respectively). Supplemental Figure 4. Correlation of average genetic expression for major cell groups shows high correlation between sample types. Fresh and cryopreserved samples correlated significantly within cell groups including keratinocytes, T/NK cells, DC/macrophages, fibroblasts, and pericytes even without filtering and normalization. Each point on the correlation plots display the average UMI counts for each gene across all cells for each major cell group. Supplemental Figure 5. Gene expression p [file 13075_2020_2343_MOESM1_ESM.zip › Supp. Figure 4.jpg]

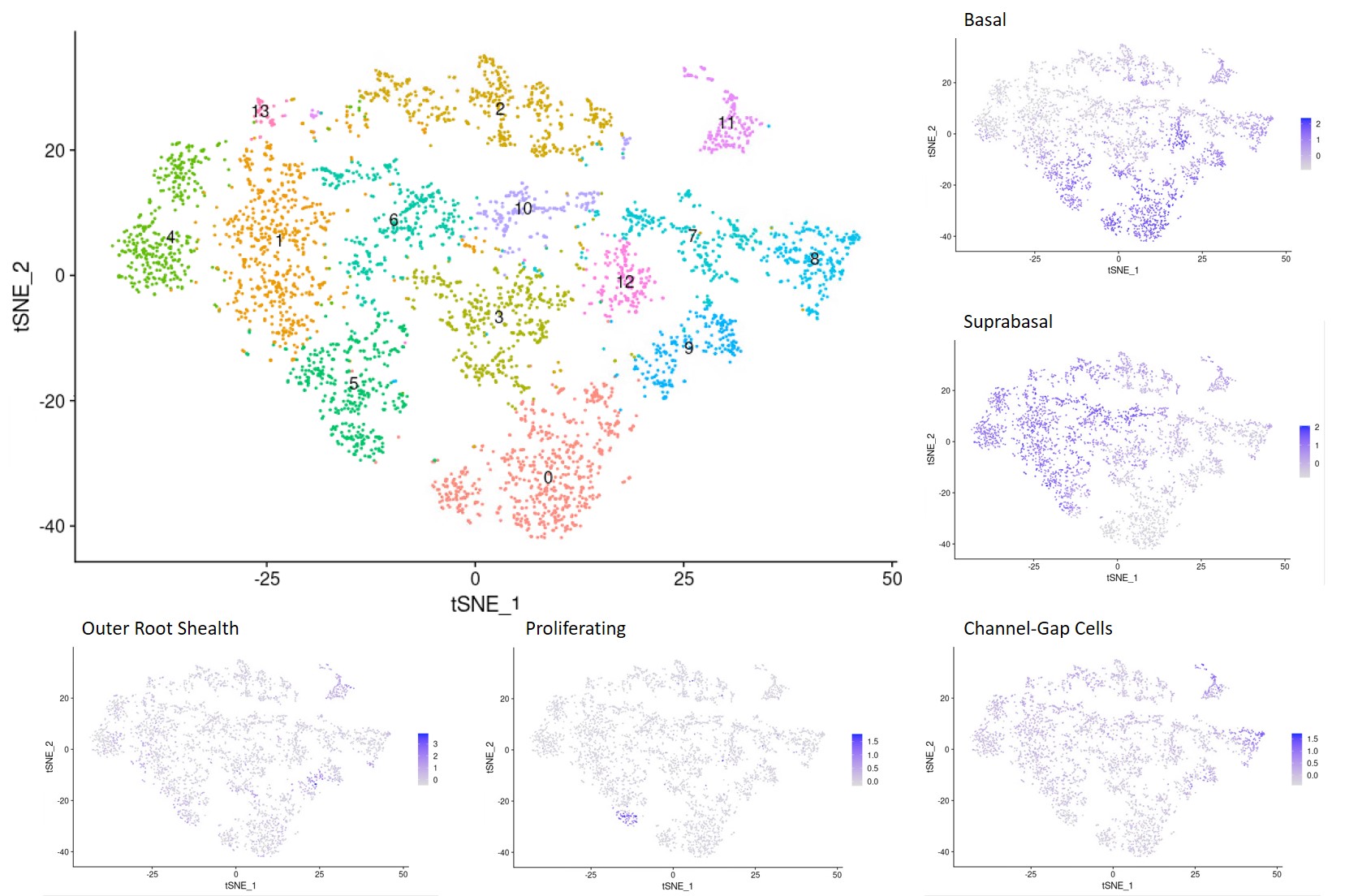

Supplement: Supplementary file 1 — Additional file 1 : Supplemental Figure 1. Quality control (QC) metrics of single cell data between cryopreserved (Cryostor CS10®, pink) and fresh (RPMI, blue) samples before filtering techniques are applied. QC metrics, including A) the number of unique genes, B) the number of total molecules, and C) the percentage of reads that map to the mitochondrial genome, all demonstrate equivalence between preservation methods before filtering and normalization with D) patient representation between clusters from three patients demonstrated (P1 – SC222, SC223, P2 – SC267, SC268 and P3 – SC272, SC273; Cryostor and RPMI samples respectively). Supplemental Figure 2. Heat map of single cell data clustering of combined cryopreserved (Cryostor CS10®) and fresh (RPMI) samples after filtering techniques are applied. Graph shows the top 5 expressed genes per the 9 identified cell groupings in the dataset. Supplemental Figure 3. Without filtering methods, samples maintain even disbursement with clustering via t-Distributed stochastic neighbor embedding (t-SNE). A) Three patients overlap well with cellular transcriptomic expression across the cell clusters. B) Cryostor® (frozen) and RPMI (fresh) preservation methods show even dispersion across cell clusters. C) Individual patient with paired frozen and fresh specimens demonstrate even dispersion. These t-SNE plots represent 15,910 skin cells, derived from 3 patients with LS (3 fresh and 3 cryopreserved samples with 9245 and 6665 cells respectively). Supplemental Figure 4. Correlation of average genetic expression for major cell groups shows high correlation between sample types. Fresh and cryopreserved samples correlated significantly within cell groups including keratinocytes, T/NK cells, DC/macrophages, fibroblasts, and pericytes even without filtering and normalization. Each point on the correlation plots display the average UMI counts for each gene across all cells for each major cell group. Supplemental Figure 5. Gene expression p [file 13075_2020_2343_MOESM1_ESM.zip › Supp. Figure 5.jpg]

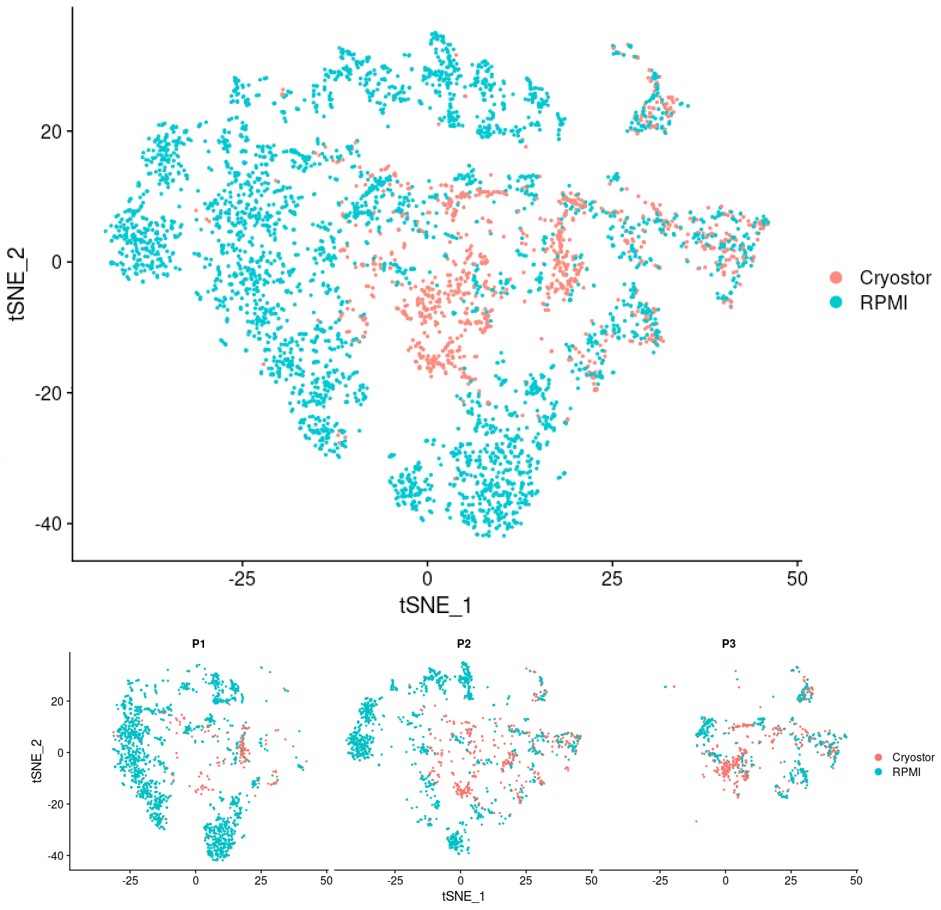

Supplement: Supplementary file 1 — Additional file 1 : Supplemental Figure 1. Quality control (QC) metrics of single cell data between cryopreserved (Cryostor CS10®, pink) and fresh (RPMI, blue) samples before filtering techniques are applied. QC metrics, including A) the number of unique genes, B) the number of total molecules, and C) the percentage of reads that map to the mitochondrial genome, all demonstrate equivalence between preservation methods before filtering and normalization with D) patient representation between clusters from three patients demonstrated (P1 – SC222, SC223, P2 – SC267, SC268 and P3 – SC272, SC273; Cryostor and RPMI samples respectively). Supplemental Figure 2. Heat map of single cell data clustering of combined cryopreserved (Cryostor CS10®) and fresh (RPMI) samples after filtering techniques are applied. Graph shows the top 5 expressed genes per the 9 identified cell groupings in the dataset. Supplemental Figure 3. Without filtering methods, samples maintain even disbursement with clustering via t-Distributed stochastic neighbor embedding (t-SNE). A) Three patients overlap well with cellular transcriptomic expression across the cell clusters. B) Cryostor® (frozen) and RPMI (fresh) preservation methods show even dispersion across cell clusters. C) Individual patient with paired frozen and fresh specimens demonstrate even dispersion. These t-SNE plots represent 15,910 skin cells, derived from 3 patients with LS (3 fresh and 3 cryopreserved samples with 9245 and 6665 cells respectively). Supplemental Figure 4. Correlation of average genetic expression for major cell groups shows high correlation between sample types. Fresh and cryopreserved samples correlated significantly within cell groups including keratinocytes, T/NK cells, DC/macrophages, fibroblasts, and pericytes even without filtering and normalization. Each point on the correlation plots display the average UMI counts for each gene across all cells for each major cell group. Supplemental Figure 5. Gene expression p [file 13075_2020_2343_MOESM1_ESM.zip › Supp. Figure 6.jpg]
